# Cancer-Induced Alterations of NK-Mediated Target Recognition: Current and Investigational Pharmacological Strategies Aiming at Restoring NK-Mediated Anti-Tumor Activity

**DOI:** 10.3389/fimmu.2014.00122

**Published:** 2014-03-24

**Authors:** Anne-Sophie Chretien, Aude Le Roy, Norbert Vey, Thomas Prebet, Didier Blaise, Cyril Fauriat, Daniel Olive

**Affiliations:** ^1^Centre de Cancérologie de Marseille, INSERM, U1068, Institut Paoli-Calmettes, Aix-Marseille Université, UM 105, CNRS, UMR7258, Marseille, France; ^2^Centre de Cancérologie de Marseille, Plateforme d’Immunomonitoring en Cancérologie, INSERM, U1068, Institut Paoli-Calmettes, Aix-Marseille Université, UM 105, CNRS, UMR7258, Marseille, France; ^3^Département d’Hématologie, Institut Paoli-Calmettes, Marseille, France; ^4^Unité de Transplantation et de Thérapie Cellulaire, Institut Paoli-Calmettes, Marseille, France

**Keywords:** cancer, immune escape, NK cell, NCR, NKG2D, KIR, immunotherapy

## Abstract

Despite evidence of cancer immune-surveillance, which plays a key role in tumor rejection, cancer cells can escape immune recognition through different mechanisms. Thus, evasion to Natural killer (NK) cell-mediated anti-tumor activity is commonly described and is mediated by various mechanisms, mainly cancer cell-induced down-regulation of NK-activating receptors (NCRs, NKG2D, DNAM-1, and CD16) as well as up-regulation of inhibitory receptors (killer-cell immunoglobulin-like receptors, KIRs, NKG2A). Alterations of NK cells lead to an impaired recognition of tumor cells as well as a decreased ability to interact with immune cells. Alternatively, cancer cells downregulate expression of ligands for NK cell-activating receptors and up-regulate expression of the ligands for inhibitory receptors. A better knowledge of the extent and the mechanisms of these defects will allow developing pharmacological strategies to restore NK cell ability to recognize and lyse tumor cells. Combining conventional chemotherapy and immune modulation is a promising approach likely to improve clinical outcome in diverse neoplastic malignancies. Here, we overview experimental approaches as well as strategies already available in the clinics that restore NK cell functionality. Yet successful cancer therapies based on the manipulation of NK cell already have shown efficacy in the context of hematologic malignancies. Additionally, the ability of cytotoxic agents to increase susceptibility of tumors to NK cell lysis has been studied and may require improvement to maximize this effect. More recently, new strategies were developed to specifically restore NK cell phenotype or to stimulate NK cell functions. Overall, pharmacological immune modulation trends to be integrated in therapeutic strategies and should improve anti-tumor effects of conventional cancer therapy.

## Introduction

Natural killer (NK) cells are key components of the innate immunity and substantially contribute to anti-tumor immune responses (1–3). The role of NK cells in immune surveillance is linked to many aspects of the NK cell biology. First, NK cells directly recognize and lyse cancer cells. Besides this direct effect, NK cells are also able to initiate anti-tumor immune responses via the secretion of various cytokines such as IFN-γ and TNF-α (1, 4).

Triggering of effector functions of NK cells is the result of a balance between activating and inhibitory signals provided by a large set of activating or inhibitory receptors. The most commonly described activating receptors involved in anti-tumor immunity are NKG2D, DNAM-1, and the natural cytotoxic receptors (NCR), NKp30, NKp44, and NKp46. Hence, NCR are NK-activating receptors of primary importance in immune surveillance and response in the context of cancer (5–7). NKp30, NKp46 are expressed by all NK cells, whereas NKp44 is only expressed by activated NK cells (8–11). The acquisition of NCR during NK cell maturation correlates with the acquisition of cytolytic activity against tumor target cells (12). NKG2D is an activating receptor also expressed by, but not restricted to, all NK cells. Ligands for NKG2D include proteins related to non-classical HLA-I such as MICA, MICB, or the structurally related ULBP1–6 (13, 14). Inhibitory receptors belong to the killer-cell immunoglobulin-like receptors (KIRs) or to the C-type lectin CD94/NKG2A heterodimer (15). These receptors recognize HLA-I and the non-classical HLA-E and inhibit NK cell activation.

The fundamental role of NK cells in oncology has been widely demonstrated in both hematologic and solid neoplasms. The relevance of this concept is illustrated by many examples in clinical practice, such as the success of hematopoietic stem cell transplantation in hematologic malignancies (16–19), poor NK cell functions associated with increased incidence of cancer (20), the importance of NK cells for the response to chemotherapy and radiotherapy (21, 22), or the use of parameters related to NK cell functions as prognostic biomarkers (23–25). Thus, NK cells can be used as prognostic biomarkers, as well as therapeutic targets or therapeutic agents.

However, although NK cells can kill target cells spontaneously without prior stimulation, a delicate balance between inhibitory and activating signals tightly regulates their activation (1, 26). In the context of cancer, this balance is often deregulated through various mechanisms (27). First of all, cancer cells are able to induce a down-regulation of activating receptors (notably NCR and NKG2D,) as well as an up-regulation of the NK cell inhibitory receptors (23, 24, 28, 29). Then, tumor cells usually poorly express ligands for activating receptors, and/or overexpress ligands for inhibitory receptors (30–32). Finally, the release of various factors such as cytokines or reactive oxygen species (ROS) within the tumor microenvironment impairs the crosstalk between NK cells and dendritic cells (DCs), enhancing the phenomenon of tumor escape (33–35).

**Figure 1:**
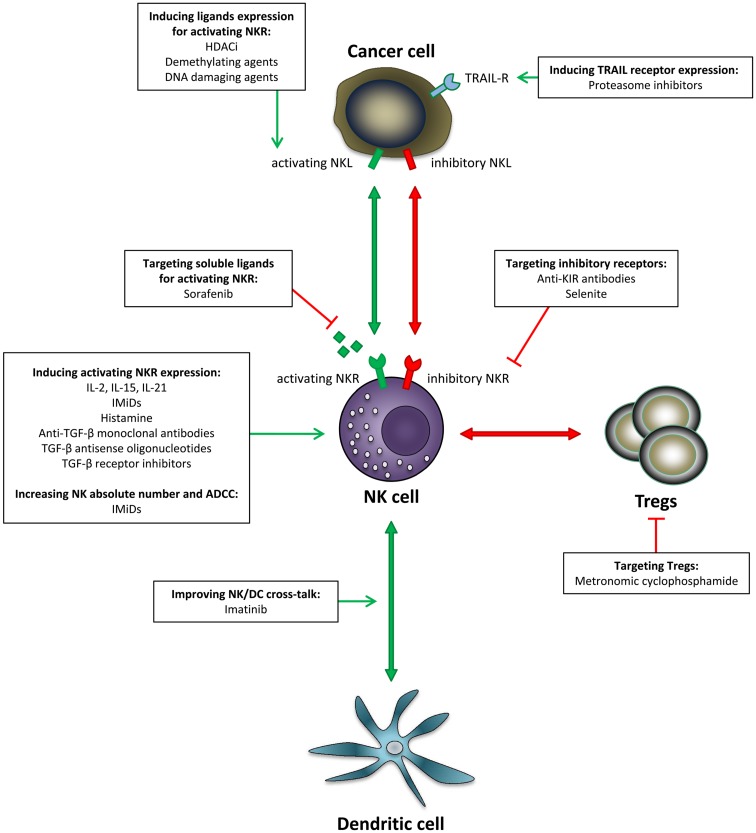
**Pharmacological strategies aiming at improving NK anti-tumor functions**. Various options have been developed to restore NK cell functionality in cancer: induction of NK triggering receptors, induction of NK ligands expression on the target cells, blockade of inhibitory signals, as well as stimulation of NK/DC crosstalk. In addition, increasing NK number and improving ADCC can enhance this effect. NKL, natural killer ligand; NKR, natural killer receptor.

Many efforts have been developed in the past few years to restore NK cell functionality in cancer patients. In this review, we focus on NK cells as a cornerstone to restore or improve anti-tumor immunity. We overview different pharmacological strategies aiming at counteracting the effect of tumor cells on NK cell functionality (Figure 1). Taking into account the crucial importance of NK cells for maintenance of a prolonged response to treatment, therapeutic strategies improving or restoring NK cell functions in combination with standard treatment regimens are expected to broadly impact patients’ clinical outcome.

## Inducing Natural Cytotoxic Receptors Expression

Natural cytotoxic receptors expression is classically downregulated during cancer progression, regardless of the type of cancer (23, 24, 28, 29). The mechanisms involved in NCR down-regulation still need to be further defined. Restoring NCR expression may render NK cells more efficient against tumor cells. So far, clinical strategies aiming at restoring NCR expression remain to be proposed. However, taking into account the strong prognostic value of NCR expression, therapeutic strategies aiming at inducing their expression is expected to improve clinical outcome. Therefore, targeting events interfering with the expression of these receptors is certainly a relevant therapeutic option (23, 25). Among possible mechanisms, Transforming Growth Factor beta 1 (TGF-β1) downregulates NKp30 and NKG2D expression on NK cells, leading to a decreased ability of NK cells to kill target cells (23, 36–38). The release of TGF-β1 is done either by the tumor cell or by regulatory T cells (Tregs). Other tumor-released soluble factors are involved in NCR down-regulation, such as Activin-A, indoleamine dioxygenase (IDO), or prostaglandin E2 (PGE2) (34, 39, 40). Similarly to other activating receptors defect, the down modulation of NCR is somehow dependent on the pressure exerted by tumor cells, which reflects a pathway for tumor evasion. Hence, in acute myeloid leukemia (AML) patients, the low NCR expression acquired during leukemia development is restored in patients achieving complete remission (23). Some recently published data suggest that NCR down-regulation is consecutive to NK activation in the tumor, leading to an exhaustion of the NK cells and a subsequent down-regulation of the NCRs (41).

### Cytokines

Amongst the efficient ways to improve NCR expression on NK cells, the use of cytokines, mainly IL-2, IL-15, and IL-21, may be promising. NK cell differentiation is cytokine-dependent (29). High baseline levels of circulating IL-2 constitute an independent prognostic factor for head and neck cancer patients (42).

#### IL-2

IL-2 is FDA-approved for cancer indications, which is not the case for IL-15 and IL-21. Most clinical trials using cytokines alone or in combination with chemotherapy or radiotherapy are set with IL-2. Conclusions of clinical trials report modest anti-tumor activity when used in monotherapy. Among its diverse immunostimulatory potentials, IL-2 is able to induce expression of NKG2D and NKp46 on NK cells (43, 44). However, following IL-2 stimulation, the NK cytolytic functions do not seem to reach normal cytolytic activity when compared to healthy volunteers (44). Moreover, IL-2 fails to induce NK cell proliferation compared to healthy volunteers, and increases the rate of apoptotic NK cells (44). Some authors evidenced the critical role of IL-2 for the development and peripheral expansion of regulatory T cells (45), which is not the case for IL-15 and IL-21. Noteworthy, the use of IL-2, especially at high doses, might be limited to *ex vivo* expansion of NK cells for problems of *in vivo* toxicity (46).

#### IL-15

IL-15 plays a major role in the proliferation, differentiation, survival, and functions of T and NK cells (29, 47). Exposure of NK cells to low doses of IL-15 significantly improved NKp30, NKp46, NKG2D, and NKG2C surface expression. Accordingly, this increase of receptor expression was correlated with an increase of natural cytotoxicity against autologous AML blasts (29, 48). In addition, in hematologic malignancies, low levels of circulating IL-15 after bone marrow transplantation were predictive of risk of relapse (49). In line, NK cell recovery in stem cell transplantation is strongly correlated with plasmatic concentrations of IL-15 (48).

IL-15 serum concentration increases dramatically following administration of cytotoxic agents (29, 49). For some authors, this elevation of serum IL-15 could be related to the depletion of lymphoid populations that normally consume circulating IL-15 or to inflammation induced by chemotherapy (48). *In vivo*, injections of the IL-15/IL-15Rα heterodimer result in significant expansion of γδ, CD8^+^ T, and NK cells (47). Recently, this cytokine has become available for use in early phase clinical trials as an alternative to IL-2 (29, 47). IL-15 is currently assessed as a therapy for various solid tumors including refractory metastatic melanoma, metastatic renal cell cancer. IL-15 is also assessed as an adjuvant of chemotherapy and vaccines strategies or prior to stem cell therapy and NK cells infusion.

#### IL-21

IL-21 shares significant structural homology with IL-2 and IL-15 (50). In phase I trials, this cytokine shows a favorable safety profile and signs of clinical activity (51). Although some reports demonstrated a deleterious effect of IL-21 by reducing activating receptor expression (NKG2D, NKp44), its main effect is to enhance NK cell functions. Hence, IL-21 is capable of inducing NK cell maturation and NKp46 and NKp30 expression (12, 52, 53). *Ex vivo*, IL-21 stimulates the production of IFN-γ and cytotoxic properties of NK cells (53). Several clinical trials reported the effect of IL-21 therapy on immune system after administration in patients with metastatic melanoma and renal cell carcinoma (51). Although NK and T-cell numbers were temporarily decreased during administration of IL-21, the cells had higher expression of CXCR3, HMMR, IFN-γ, perforin, and granzymes at the mRNA level. Evidence of NK cell activation was further confirmed by enhanced ability of NK cells from patients to lyse K562 target cells (51). These results were confirmed in a phase II trial for metastatic melanoma (54).

### Immunomodulatory drugs

Immunomodulatory drugs (IMiDs) present another therapeutic option to increase activating receptors expression. Two molecules are currently developed in oncology: lenalidomide, FDA-approved in hematologic malignancies, and pomalidomide. These drugs present anti-angiogenic and anti-proliferative activity, and their effect on the immune system, particularly on NK cells, is probably part of their mechanism of action. For instance, immunomonitoring of patients treated with immunomodulatory drugs, IMiDs have been associated with an increased expression of NKp44 and NKp46, in multiple myeloma (MM), myelodysplastic syndrome but also in solid tumors (55, 56). Interestingly, this effect of lenalidomide may not be a direct effect on NK cells because this effect was not observed *in vitro* on purified NK cells (57). In this study, IMiDs-treated NK cells displayed a lower NKp46 expression, although this had no functional consequences on cytolytic functions of NK cells.

### Histamine

Blocking phenomenon responsible for NCR down-regulation is another potential strategy to induce indirect NCR expression. Thus, ROS, PGE2, and IDO, which are present in the tumor microenvironment, appear to be relevant targets (33–35). Romero et al. demonstrated that histamine was able to prevent NKp46 and NKG2D down-regulation mediated by mononuclear and polymorphonuclear phagocytes ROS production (35). Moreover, histamine maintains the cytolytic activity of NK cells toward leukemic cells despite the presence of phagocytes. A phase III clinical trial assessed the efficacy of post-consolidation immunotherapy with IL-2 and histamine dihydrochloride for patients with AML in complete remission. This treatment was shown to significantly improve leukemia-free survival, with mild to moderate side effects (33).

## Inducing NKG2D Expression

NKG2D down-regulation on circulating NK cells in cancer patients compared to healthy volunteers was described in various cancer types, including breast cancer, glioma, melanoma, and lung cancer (58–62).

### Cytokines

Few pharmacological agents are able to directly increase the expression of NK-activating receptors. Until now, the only described possibility to directly induce NKG2D expression on NK cells is the use of immunostimulatory cytokines. *Ex vivo*, IL-15 was shown to be able to induce a dramatic increase of NKG2D expression (63, 64). Although the use of IL-15 is still restricted to phase I and II clinical trials, conventional chemotherapies are able to induce a huge increase of the circulating IL-15 (29).

### TGF-β pathway

A second strategy allowing NKG2D restoration in the cancer context is indirect up-regulation by blocking the agents responsible for NKG2D down-regulation. For instance, stroma-derived factors in the tumor microenvironment, in particular TGF-β, display an immunosuppressive activity on most anti-tumor immune effectors, and an indirect immunosuppressive effect via the inhibition of MICA transcription (38, 65). Besides immune suppression, stroma-derived factors also present direct effects on the tumor cell since TGF-β promotes tumorigenesis and epithelial–mesenchymal transition (66). *In vitro*, TGF-β inhibits the expression of NKp30 and NKG2D (37) and blood concentration of TGF-β1 was shown to inversely correlate with NKG2D expression at the surface of NK cells of cancer patients and has been linked with impaired NK cytotoxicity (58, 60). TGF-β antagonizes the IL-15-induced proliferation and gene expression associated with NK cell activation, inhibiting the expression of NK cell activation receptor molecules (67). Moreover, *ex vivo* addition of neutralizing anti-TGF-β monoclonal antibodies completely restores surface NKG2D expression at the surface of NK cells and partially restores NKp30 expression (60, 67). In addition, blocking TGF-β completely restores IFN-γ production by tumor-associated NK cells (67).

Some approaches aiming at decreasing circulating TGF-β in patients are currently under investigation (68). These early stage clinical trials currently assess several approaches, mainly the use of anti-TGF-β monoclonal antibodies and antisense oligonucleotides. For example, fresolimumab (GC-1008), a fully humanized pan-neutralizing antibody directed against all the three isoforms of TGF-β, has been assessed in renal cell carcinoma and in metastatic melanoma (68, 69). In this phase I/II trial, fresolimumab was safe and well-tolerated with no dose-limiting toxicities and displayed encouraging results.

The impact of TGF-β blockade on immune parameters was recently assessed in patients with malignant pleural mesothelioma treated with fresolimumab (70). Fresolimumab had no effect in the expression of NK, CD4^+^, or CD8^+^ T-cell-activating and inhibitory markers, other than a decrease in the expression of 2B4 and DNAM-1 on NK cells, although TGF-β serum concentrations were markedly decreased. The authors conclude that acute changes in serum TGF-β concentration are not associated with the set of biomarker changes that were predicted based on animal models. No effect was detected on the expression of NKG2D nor NKp30, and the effect on DNAM-1 expression, although significant, was minor (70).

Another possibility to decrease TGF-β in the tumor milieu is the use of antisense oligonucleotides. Some of these compounds are currently in clinical evaluation. Belagenpumatucel-L, a therapeutic vaccine comprised of four TGF-β2 antisense gene-modified allogeneic NSCLC cell lines was assessed in grade III/IV NSCLC patients. In a phase II study, positive clinical outcomes were correlated with immune response to the vaccine and induction of immune enhancement of tumor antigen, but the effect on NK cells was not assessed (65). This compound is still currently investigated in non-small cell lung carcinoma in phases II and III trials.

Alternatively, SD-208, a TGF-β receptor I kinase inhibitors, restores the lytic activity of polyclonal NK cells against glioma cells in the presence of recombinant TGF-β or of TGF-β-containing glioma cell supernatant (71). This molecule is able to restore NKG2D expression on NK cells, whose expression was altered *in vitro* by cancer cell lines supernatants or direct inhibition with recombinant TGF-β (72).

To conclude, NKG2D expression has never been shown to present a prognostic value unlike NKG2D ligands expression, thus suggesting that the best strategy to target the NKG2D/NKG2D ligand system might be to induce ligands expression rather than the receptor itself.

## Inducing Ligands Expression for NK-Activating Receptors

The main ligands for NKG2D are the MHC class I chain-related molecules MICA and MICB and the ULBP1–4. These ligands have been extensively studied in various malignancies. Ligands of DNAM-1 are CD112 (Nectin-2) and CD155 (Poliovirus receptor, PVR). Ligands of NCRs have been elusive for many years and although pathogen-related ligands have been suggested (hemagglutinins, heparate sulfates), only ligands for NKp30 have been identified. B7-H6, an Ig molecule from the family of B7 molecules has been identified as NKp30 ligand (73). B7-H6 is expressed by several cell lines and by primary tumors (74). Mechanisms of induction of B7-H6 expression have been described in non-transformed cells with TLR agonists as well as the pro-inflammatory cytokines TNFα and IL-1β (75). In primary tumors, recent experimental data suggest that B7-H6 expression is regulated by HDACs, in particular HDAC3 (74). In addition, BAG6/BAT3, a nuclear protein localized at the plasma membrane or on exosomes of tumor cells, has also been assigned as an NKp30 ligand (76). The importance of ligands expression for tumor cell recognition by NK cells is a key factor for anti-tumor immune response, as illustrated by the strong prognostic value of MICA/MICB, RAET1G, and ULBP2 expression in colorectal cancer and breast cancer (30–32). Tumor cells poorly express ligands for NK-activating receptors, and tumor ligands expression is inversely correlated with clinical stage (77).

### Histone deacetylase inhibitors

Histone deacetylase inhibitors were successfully introduced as anti-cancer agents for their ability to block gene transcription and promote cell differentiation. These molecules induce cell cycle arrest and induce apoptosis of tumor cells, with minimal effects on normal tissue (78). Unexpectedly, their effect on anti-tumor immunity is part of their mechanism of action.

The main impact of these molecules on immunity is mediated through up-regulation of tumor antigens, in particular NKG2D ligands (79). HDACi-mediated immune modulation is also linked to the ability of these molecules to enhance immune recognition and lysis of the tumor cells by T cells and NK cells (79). To date, two molecules, romidepsin and vorinostat, have received approval from the FDA for the treatment of cutaneous T-cell lymphoma. *In vitro*, romidepsin, vorinostat, and sodium valproate were shown to increase MICA/B and ULBPs expression on various cancer cell lines and primary tumor cells, and render the target cells more sensitive to NK cell lysis (80–84). Depending on the authors, this mechanism was found to be GSK3- or ERK-dependent (81, 83).

Induction of MICA and MICB expression was associated with a shedding of the soluble forms of these NKG2D ligands, sMICA and sMICB (82). This raises the question of the potential counterbalancing of the clinical benefits in this particular case, since increase of the serum concentrations of sMICA and sMICB are responsible for NKG2D endocytosis and degradation, and represents a mode of T-cell silencing and immune escape (62, 82). Thus, Poggi et al. monitored NKG2D ligands shedding following treatment of AML patients treated with valproic acid. In this study, MICA, ULBP2, and ULBP3 expression on blasts was significantly increased after treatment with valproic acid. No ligand shedding was detected despite a strong up-regulation of the ligands on leukemic cells. Consequently, leukemic cells from patients treated with valproic acid, become able to trigger lytic granule exocytosis by autologous CD8^+^ T and NK cells (85).

However, some studies evidenced that HDACi down-regulate ligands for other NK cells-activating receptors, such as B7-H6, a ligand for NKp30, and impair tumor cell recognition by NK cells. These results were obtained with first and second generation HDACi (vorinostat, trichostatin A, valproic acid, and apicidin) on various cancer cell lines (74). Moreover, treatment of human NK cells with trichostatin A, valproic acid, or sodium butyrate affects the functional response of human NK cells, evidenced by a strong inhibition of IFN-γ secretion and a decreased ability to lyse target cells (86). Furthermore, the authors evidenced a down-regulation of activating receptors NKG2D and NCRs on resting and cytokine-stimulated NK cells.

Another study assessed the effect of vorinostat and valproic acid on NK cells. At therapeutic concentration, these drugs induced the down-regulation of NKp30 and NKp46, and inhibited IL-2 activation of NK cells, thus suppressing their cytolytic activity toward leukemic cell lines. This effect seems to be mediated by the inhibition of NFκB. In addition, the authors showed that vorinostat was toxic to NK cells in the range of therapeutic concentrations (87).

### Demethylating agents

The hypomethylating drugs decitabine and azacytidine are epigenetic drugs that are currently used in treatment of hematological malignancies (88). Besides their direct effect on the tumor cell, these drugs probably act through their impact on innate immunity. *In vitro*, both drugs induce ULBP1 and MICB on cell lines and primary tumor cells when incubated with either decitabine or 5-azacytidine (89, 90). This effect was related to promoter DNA methylation and DNA damage and correlates with enhanced NK cytotoxicity (90, 91).

However, DNA methylation is an important regulator of KIR expression by NK cells, potentially impacting on NK cell functions (92, 93). Hence, 5-azacytidine induces an increase in the percentage of KIR^+^ NK cells upon treatment with clinically relevant concentrations of 5-azacytidine, which correlated with an impaired granzyme B and perforin release, IFN-γ production, and decreased cytotoxicity (91, 94). However, this effect seems to be restricted to 5-azacytidine, since decitabine increases NK cell cytotoxicity and enhances IFN-γ production, in a dose-dependent manner (91). These results were confirmed in recent studies in different settings. Recently, Cerdeira et al. tested the effect of 5-azacytidine in hypoxic conditions with addition of TGF-β. Although the authors confirmed the impact of this drug on KIR expression, however, the cytotoxicity of NK cells cultured in these specific conditions was not affected (92).

For some authors, the results obtained *in vitro* in such settings are debatable. Indeed, since 5-azacytidine and decitabine are nucleoside analogs, these molecules require DNA replication to be incorporated into the DNA strand. *In vitro* studies using resting NK cells are therefore more likely to reflect the direct mRNA effect of such drugs than the effect of hypomethylation (88). Thus, Kopp et al. studied the effect of decitabine on proliferating NK cells. The authors show that decitabine negatively affects NK cell viability and proliferation in a dose-dependent manner. Simultaneous increase in KIR and NKp44 expression and decrease in NKG2D expression was evidenced. However, the impact on NK functionality in terms of toxicity was biphasic, with decreased toxicity at low doses and increased toxicity at high doses. Since the target cells used in these experiments lack class I HLA, this effect is independent of KIR up-regulation. Whether this increased cytotoxicity is maintained in the presence of HLA-positive targets remains to be determinate (88).

To conclude, further investigation is required to determine whether epigenetic drugs adversely affect NK cell survival, proliferation, or functions when administrated to patients.

### DNA-damaging agents

Some conventional chemotherapeutic agents can induce immunogenic cell death, e.g., tumor cell apoptosis and stress signals that lead to the surface expression of ligands for NKG2D and DNAM-1 (95, 96). This DNA damage pathway can be activated by several mechanisms, during the course of chemotherapy with DNA-damaging agents such as doxorubicin, mitoxantrone, cisplatin, and oxaliplatin (8, 95–99). This particular mode of cell death displays damage-associated molecular patterns, e.g., exposure of calreticulin endoplasmic reticulum proteins at the surface of the pre-apoptotic cell, as well as secretion of ATP (100).

The oncogenic stress induced by these DNA-damaging agents stimulates various aspects of anti-cancer immunity, including activation of NK cells via ULBP1, MICA/B, and PVR expression at the surface of the cancer cell in an ATM (ataxia telangiectasia, mutated), ATR (ATM- and Rad3-related) protein kinases, and/or P53-dependent manner (8, 96–99). Other agents are able to induce stress conditions, leading to the expression of ligands for NKG2D and DNAM-1, such as IMiDs and proteasome inhibitors (22). These results await clinical confirmation with immunomonitoring studies of patients undergoing DNA-damaging agent therapy.

## Targeting Soluble Ligands for Activating Receptors

The expression of NKR ligands at the surface of cancer cells appears to be a good prognostic factor. However, the shedding of soluble ligands in the circulation strongly impairs NK cell functions and has been linked with tumorigenesis and tumor progression (101) and high serum concentration of ULBP2 presents a strong prognostic value in breast cancer, colorectal cancer, and melanoma (30–32). Noteworthy, the discovery of B7-H6 and BAG6, ligands for NKp30, included the detection of soluble forms, which may compete for cell–cell interaction with membrane-bound ligands, although only soluble/exosome-bound BAG6 has been detected in a cancer situation (75, 102).

The prototypical example of ligand shedding is the release of soluble MICA/MICB (sMICA/sMICB), typically by A disintegrin and metalloproteases (ADAMs) (103, 104). These proteases are overexpressed in malignant tissues compared to normal tissues (105, 106). As a consequence, serum concentrations of soluble ligands for NKG2D are elevated in various malignant conditions (103). The ligation of these soluble ligands induces internalization of NKG2D and its subsequent degradation, leading to an overall down-regulation of the receptor at the surface of NK cells. In various cancers, high levels of circulating ligands for NK-activating receptors correlated with a poor prognosis. Direct pharmacologic inhibition of these metalloproteases is still in preclinical evaluation.

### Sorafenib

Sorafenib is a multi-target tyrosine kinase inhibitor targeting RAS/RAF/MAPK as well as VEGFR and PDGFR signaling pathways, implicated in cell proliferation and angiogenesis. Sorafenib is indicated in renal cell carcinoma, hepatocellular carcinoma, thyroid cancer and melanoma. *In vitro*, this molecule presents interesting off-target effects on ADAM9 expression as evidenced by a recent study on the human hepatocellular carcinoma cell line HepG2. In this study, sorafenib was able to strongly decrease ADAM9 expression at the proteic and transcriptional level, which correlated with a decrease of sMICA concentration in the culture supernatant and enhanced sensitivity to NK cell lysis. In addition, ADAM9 inhibition increases the expression of membrane-bound MICA on the tumor cell, enhancing the NK sensitivity of hepatocellular carcinoma cells (105).

Controversial data were published about effects of sorafenib on NK cells. NK cell function is inhibited by sorafenib as a consequence of impaired phosphorylation of PI3K and ERK, which directly control NK cell reactivity (107). Immunomonitoring of patients with renal cell carcinoma and melanoma treated with sorafenib failed to evidence modification of pERK1/2 expression in peripheral-blood NK cells after short-term or long-term administration (108). In addition, sorafenib may also positively (Th1) or negatively (DCs) impact other aspects of anti-tumor immunity (61, 109, 110). Whether this action is positive or negative remains to be determinated, as well as the overall “immune benefit” of such antagonistic effects on anti-tumor immunity, besides their direct pro-apoptotic effect on the tumor cell.

## Targeting Inhibitory Receptors

Although activating NK receptors are crucial, triggering of NK cell effector functions is prevented by the expression of the inhibitory receptors KIR and NKG2A. Although in some examples of solid cancer, KIR and NKG2A expression is altered, generally expression is maintained and tumor cells may maintain sufficient amounts of HLA molecules to ensure inhibition of NK cells and evade killing. Moreover, some tumors display decreased expression of TCR-dependent HLA molecules while maintaining a normal expression of KIR-dependent HLA molecules (111). High HLA-E expression has been observed in several solid cancers (112, 113) and leukemias (114). Consequently, as 20–70% of NK cells express NKG2A, HLA-E expression by tumor cells impairs the anti-tumor activity of a predominant proportion of NK cells.

### Anti-KIR monoclonal antibodies

Among the strategies to improve the recognition of tumor cells by NK cells, blocking the inhibitory interactions is appealing. The most advanced therapeutic compound as for today is the anti-KIR monoclonal antibody, IPH2101. This fully humanized antibody blocks the interaction of the major KIR expressed by NK cells with their cognate ligands, i.e., HLA-C. This reagent has been tested in early phase clinical trials and was shown to be well-tolerated in patients suffering from AML (115). In some instance, NK cells from treated patients expressed the activation marker CD69 and IFN-γ or MIP-1β was detected in the sera of patients. Another clinical trial in patients with MM has also shown that IPH2101 is safe and also enhances *ex vivo* NK cell cytotoxicity against MM cells (116). IPH2101 (and its replacement IPH2102) is therefore a novel immune-therapeutic agent that may improve anti-tumor activity of patients. More trials are programed and already necessary but yet this reagent has reached the promises for clinical use against cancer cells.

### Selenite

As mentioned above, control of NK cell activation is either achieved by KIR/HLA interactions but also NKG2A/HLA-E interaction. In healthy individuals, at steady state, the two systems compensate for each other to ensure a total control of NK cell reactivity. Regarding NKG2A-mediated inhibition of NK cells by HLA-E expressing tumor cells, very few data are available. Interestingly, an FDA-approved reagent, selenium, may be a promising tool. Supplementation with selenium has been associated with reduced risk of solid cancer (117). The mechanism of action of selenium is not entirely known, but it induces apoptosis of tumor cells by generating an oxidative stress, which may be more effective on tumor cells compared to healthy cells (118, 119). Alternatively, selenium blocks the synthesis of HLA-E and consequently increases cytotoxicity mediated by NKG2A-positive NK cells (120). This effect, combined to the direct toxicity on tumor cells may result in reduced disease progression and improved survival. Sodium selenite is currently under investigation in several clinical trials for the treatment of different cancers.

Altogether, targeting inhibitory NK receptors reflects a novel orientation taken for innovative therapeutic approaches, as it represents another way to counteract the immune escape via ligands for inhibitory receptors. Of note, this strategy relies on the expression of activating ligands by leukemic cells. Hence, removing of inhibition will allow NK cells killing their targets provided that they express the ligands for activating NK receptors.

## Alternative Pathways to Improve NK Activity

### Increasing NK cell lysis capacity with IMiDs

Immunomodulatory drugs are capable to enhance monoclonal antibodies anti-tumor activity. First *in vitro*, Wu et al. have shown an enhancement of NK cell-mediated tumor cell ADCC by lenalidomide for a variety of rituximab-treated NHL (non-Hodgkin lymphoma), cetuximab-coated CRC (colorectal cancer), and trastuzumab-coating breast cancer cell lines (121, 122). Another team highlighted the enhancement of ADCC by lenalidomide *in vitro*. They have shown an increase of Raji cell apoptosis mediated by PBMC combination with rituximab by lenalidomide (123). In the first case, the effect was observed on purified NK cell but Wu et al. have explained that this mechanism is dependent on the presence of antibody and either interleukin-2 or interleukin-12. In the second case, Zhu et al. have observed this effect on PBMC. Finally, the researches of Hayashi et al. have shown that IMiDs-enhanced NK cell ADCC by triggering IL-2 production from T cells (124). All these works suggest that *in vitro* IMiDs-positive effect on NK cell ADCC could be dependent on IL-2.

In animal models, lenalidomide or pomalidomide in combination with rituximab improves severe combined immunodeficient (SCID) lymphoma-bearing mouse survival compared to rituximab in monotherapy (125). Three years later, the same team explained this enhancement of anti-tumor activity by an expanding, activating, and trafficking of NK cells into the tumor bed, which facilitate a more efficient ADCC. The IMiDs effect on NK cells in this model is also associated with DC activation and production of chemokines and pro-inflammatory cytokines (126).

In the same way, IMiDs are also capable to enhance natural cytotoxicity of NK cell against cancer cells. First, Davies et al. highlighted the potency of thalidomide, lenalidomide, and pomalidomide to increase PBMC cytotoxicity toward MM tumor cells (cell lines and patient cells) *in vitro*. They presented this effect as an NK-dependent effect (127). Then, Zhu et al. have shown the similar effect with lenalidomide and pomalidomide on K562 and PC-3 cell lines (i.e., enhanced PBMC-mediated tumor cell apoptosis). They have also shown that NK cells are essential in inducing cancer cell apoptosis (123). In the same manner as ADCC, Hayashi et al. have explained this IMiDs enhancement of NK cell cytotoxicity via induction of IL-2 production in T cells (124).

In line with *in vitro* studies, IMiDs also increased NK cell natural cytotoxicity in patients suffering from MDS or solid tumors (56). At last, IMiDs have an important property toward NK cell numbers. Hence, the number and the localization of NK cells in cancer patients is often correlated with prognosis (24, 25, 128–130).

Davies et al. observed that thalidomide treatment for MM patients resulted in an increase of absolute NK cell numbers (127). This observation was confirmed with lenalidomide in some metastatic malignant melanoma patients and other advanced cancers (131), and in children with solid tumors or MDS (56). This effect was also highlighted in lenalidomide and pomalidomide treated mice (lymphoma-bearing SCID mice) at the tumor site. Reddy et al. have shown in their study an increase of tumor central infiltration by NK cells in mice treated by lenalidomide or pomalidomide compared to DMSO-treated mice. They could explain that by the IMiDs effects on DCs stimulation and modification of the cytokine microenvironment (126).

### Inducing TRAIL receptor expression on target cells

Proteasome inhibitors are a class of anti-cancer drugs that are used in first line of treatment of MM, and that are currently evaluated in hematologic and solid malignancies. These molecules disrupt proteasome activity, resulting in cell growth arrest, apoptosis, angiogenesis inhibition, and decreased binding of tumor cells to stromal cells (132). *In vitro*, bortezomib was shown to sensitize tumor cell lines as well as primary tumor cells to perforin/granzyme-mediated NK-tumor cytotoxicity. This effect was found to be dependent on augmentation of tumor caspase-8 activity as well as on up-regulation of Fas and TNF related apoptosis-inducing ligand (TRAIL) receptor on tumor cells, thus inducing target apoptosis by NK cells through Fas/FasL and TRAIL/DR5 interactions (133–135). Other proteasome inhibitors such as the b-A15 share this property (136). In addition, proteasome inhibitors up-regulate ULBP1 and ULBP2 expression (137–139). This effect is accompanied by a down-regulation of HLA class I molecules (140).

*In vivo*, bortezomib sensitizes tumors to killing by NK cells. This anti-tumor effect is enhanced upon depletion of Tregs (134, 141). Based on these results, a non-randomized phase I study is currently ongoing in order to evaluate the safety and the anti-tumor effects of adoptively infused *ex vivo* expanded autologous NK cells against metastatic cancers or hematological malignancies sensitized to NK TRAIL cytotoxicity with bortezomib (134). However, bortezomib paradoxically renders tumor cells resistant to killing by tumor-specific T cells, thus potentially counterbalancing the benefits obtained through the sensitization to killing by NK cells (136, 142). In addition, *in vitro* assays evidenced that bortezomib presents pro-apoptotic effects on NK cells, and induces a down-regulation of NKp46 expression with subsequent decrease in NKp46-mediated activity (143). b-AP15, a new proteasome inhibitor, appears to overcome this deleterious effect on T cells: *in vitro* evaluation of this molecule was shown to sensitize tumor cell lines to both NK and T cell-mediated killing (136). However, at equipotent doses, this molecule seems to be more toxic to NK cells than bortezomib (144).

### Improving NK/DC crosstalk

The relevance of the NK/DC crosstalk has been demonstrated in various physiopathological settings and alterations of these interactions have been shown to contribute to tumor progression (145). Imatinib mesylate is a tyrosine kinase inhibitor that inhibits the tyrosine kinase encoded by the bcr-abl oncogene and tyrosine kinases encoded by the c-kit and the PDGFR oncogenes. Targeting these tyrosine kinases directly induces apoptosis of the cancer cell, which constitutes the main mechanism of action of imatinib. Besides this direct anti-proliferative effect, an “off-target” effect, inducing DC-mediated NK activation was described by Borg et al. (145). In this study, patients with GIST were assessed for NK cell functions during the course of treatment with imatinib. Anti-tumor response correlated with enhanced NK-mediated anti-tumor response, thus bringing out a new mechanism of action of this molecule. The authors then defined immunologic responder patients with increased RFS. In a more recent study conducted in GIST patients, the authors validated the concept, showing a correlation between clinical outcome and NK cell activation induced by therapy with imatinib (21). Immunomonitoring of NK cell functions included IFN-γ production and NKG2D expression. Although IFN-γ production was associated with clinical outcome, enhanced NKG2D-dependent lysis observed at 1 year of imatinib therapy did not impact survival (21). Interestingly, this DC-mediated NK activation seems to occur in lymph nodes where imatinib promotes the formation of immunologic synapses with resting or preactivated NK cells as a consequence of the blocking of KIT signaling in DCs (21, 97).

### Depleting Tregs

Tregs inhibit antigen-specific immune response both in a cytokine-dependent and cell contact-dependent manner (146–148). Tregs alter both T cells and NK cells proliferation and activity through the down-regulation of NKG2D (147–149). Increased frequency of Treg cells and low T effector (Teff)–Treg ratios are associated with a poor clinical outcome and a lack of treatment response (147, 150–153). Impairment of Treg activity by either specific blockade or depletion can enhance immune response against tumor-associated antigens (147, 148). To date, drugs that specifically target Tregs are not available (153).

Although cyclophosphamide is immunosuppressive at high doses, this molecule displays particularly interesting immunostimulatory properties in metronomic scheduling (iterative administration of low doses) mainly by its ability to suppress FOXP3^+^ regulatory T cells (95, 149, 154) and to induce TH2/TH1 to TH17 shifts in cytokine production, induction of TH17, and resetting of dendritic cell homeostasis (153, 155). In murine models, metronomic cyclophosphamide strongly induces NKp46 expression as well as perforin and granzymes (156). Importantly, immunomonitoring studies evidenced that low-dose cyclophosphamide regimen restores patients’ T cells and NK cells functions as evidenced by killing assays (149, 157). Metronomic cyclophosphamide is currently tested in combination with anti-cancer vaccines, for its ability to suppress Tregs in order to facilitate vaccine-induced tumor rejection (153). Despite metronomic cyclophosphamide provides promising clinical results, some authors point the absence of randomization in these trials (158).

## Concluding Remarks

Accumulating evidence based on immunomonitoring analyses highlights immune parameters as strong prognostic factors, both in hematopoietic and solid neoplasms. These conclusions provide a strong rationale for developing therapeutic strategies aiming at restoring key immune parameters. Among the major mechanisms used by tumor cells to escape immunity, the evasion from receptor–ligand-mediated anti-tumor activity by NK cells represents the most prevalent pathway. Hence, the recognition of tumor cells by NK cells via NCR or NKG2D-activating receptors is often impaired in various cancers and enhancing NK cell functions appears as one of the most promising approaches. One important question remains the ability of a cancer cell to overcome immune suppression upon exposure to immunostimulating drugs. Recent studies suggest that NK cells on tumor site exhibit a phenotype of exhaustion and terminal differentiation. Restoring NK functionality in this context could be of limited interest since these cells may hardly become highly anti-tumoral. This parameter should be considered to maximize the effects of such approaches.

To conclude, targeting immune evasion mechanisms, in association with conventional chemotherapy, may improve clinical outcome and is clinically feasible with limited side effects. To date, clinical application of this concept is mainly limited to drugs designed to target cancer cells, with off-target effects on the immune system. The problem of these strategies is that the overall benefit on the different immune effectors is sometimes hard to predict, and can be deleterious on crucial immune effectors, although restoring other cells. New strategies aiming at specifically restored immune functions will be potentially more efficient, and are currently in preclinical and clinical development. Further development of these immune therapies urges to associate clinical trials with translational immunology and immunomonitoring. A better knowledge regarding immune evasion mechanisms will definitely provide the absolutely required bases for the next-generation immune cancer therapies.

## Conflict of Interest Statement

The authors declare that the research was conducted in the absence of any commercial or financial relationships that could be construed as a potential conflict of interest.
